# Managing neurofibromatosis type I and vision impairment in a resource-limited setting: a case study and multidisciplinary approach

**DOI:** 10.1097/MS9.0000000000002482

**Published:** 2024-08-26

**Authors:** Pratik Adhikari, Nabin Bhujel

**Affiliations:** a Koirala Institute of Health Sciences, Dharan; b College of Medical Sciences, Bharatpur, Chitwan, Nepal

**Keywords:** multidisciplinary care, neurofibromatosis type I (NF1), resource-limited settings, visual impairment

## Abstract

**Background:**

Neurofibromatosis type I (NF1) is a genetic disorder characterized by the development of multiple benign tumors along nerves in the skin, brain, and other parts of the body. It is associated with a range of clinical manifestations, including skin lesions, neurofibromas, and ocular abnormalities, which can significantly impact a patient’s quality of life. Management of NF1 is particularly challenging in resource-limited settings due to limited access to diagnostic and therapeutic resources.

**Clinical presentation:**

A 62-year-old woman with a known history of NF1 presented with progressive visual impairment. Her condition began in childhood with multiple hyperpigmented skin macules, which developed into numerous cutaneous tumors over time. Examination revealed numerous neurofibromas, café-au-lait spots, and axillary freckling. Significant visual impairment was caused by large fibromas on her eyelids. Histological analysis confirmed benign nerve tissue tumors.

**Clinical discussion:**

The management strategy in this resource-limited setting focused on regular monitoring, patient education, symptomatic treatment, and multidisciplinary care. Despite the limitations, the patient’s condition was managed effectively through these adapted strategies. The importance of genetic testing for confirmation and further management was noted but not performed due to resource constraints.

**Conclusion:**

This case highlights the complexities of managing NF1 in resource-limited settings, emphasizing the need for adaptable management approaches. Multidisciplinary care and patient education were crucial in improving the patient’s quality of life. This case underscores the importance of early diagnosis and intervention to prevent complications like visual impairment.

## Introduction

Highlights

- This case highlights the challenges of diagnosing and managing neurofibromatosis type I (NF1) in a resource-limited setting.
- Significant visual impairment was caused by large fibromas on the patient’s eyelids.
- Effective management included regular monitoring, patient education, and multidisciplinary care.
- The case underscores the importance of early diagnosis and adaptable management strategies to improve patient outcomes.

Neurofibromatosis type I (NF1) is an autosomal dominant genetic disorder that affects ~1 in 3000 individuals worldwide^1,2^. Characterized by multiple café-au-lait spots, neurofibromas, and Lisch nodules, NF1 is caused by mutations in the NF1 gene located on chromosome 17^3^. This gene encodes neurofibromin, a protein involved in the regulation of cell growth, which, when mutated, leads to the development of benign and malignant tumors^4^. NF1 has a broad spectrum of clinical manifestations that can significantly impact the quality of life of affected individuals^5^.

The diagnosis of NF1 is primarily clinical, relying on the criteria established by the National Institutes of Health (NIH), which include the presence of multiple café-au-lait spots, neurofibromas, Lisch nodules, and other characteristic features^6^. Recent advancements have improved our understanding of the genetic basis and pathophysiology of NF1, aiding in the development of targeted therapies and management strategies^7,8^. However, the management of NF1 remains challenging, especially in resource-limited settings where access to specialized care and advanced diagnostic tools may be restricted^9^.

This case report presents a unique and complex case of NF1 in a 62-year-old woman who developed significant visual impairment due to multiple cutaneous neurofibromas on her eyelids. The case highlights the diagnostic challenges and management strategies implemented in a resource-limited setting, emphasizing the importance of multidisciplinary care and patient education in managing NF1.

## Case presentation

A 62-year-old woman with a known history of NF1 presented with progressive visual impairment. Her chief complaints included increasing difficulty in vision due to multiple cutaneous tumors, particularly on the eyelids, and a large fibroma obstructing her peripheral vision. The patient’s family history revealed that her father also had multiple café-au-lait spots, suggesting a possible hereditary component. Additionally, the patient had a history of intellectual disability following encephalitis during childhood.

The patient reported that her condition began in childhood with the appearance of multiple hyperpigmented skin macules. Over the years, these lesions progressively increased in number and size. By the age of 44, numerous cutaneous tumors developed, predominantly on her face and trunk, which continued to grow over time. Despite the significant number of tumors, the patient did not seek medical attention earlier due to her mental retardation. Visual difficulties prompted her to seek care eventually, particularly due to large tumors on her eyelids, causing partial ptosis and restricted peripheral vision.

On physical examination, the patient’s vital signs were stable, with a blood pressure of 130/80 mmHg, a pulse rate of 78 beats per min, a respiratory rate of 18 breaths per minute, and an SpO2 of 98% on room air. Systematic examination revealed normal findings across multiple systems: cardiovascular examination showed normal heart sounds with no murmurs; respiratory examination revealed clear lung fields with no abnormal breath sounds; abdominal examination was unremarkable, with a soft and non-tender abdomen and no organomegaly; and central nervous system examination indicated that the patient was alert, albeit with mild intellectual disability, and no focal neurological deficits were observed.

## Dermatological status

**Figure 1:**
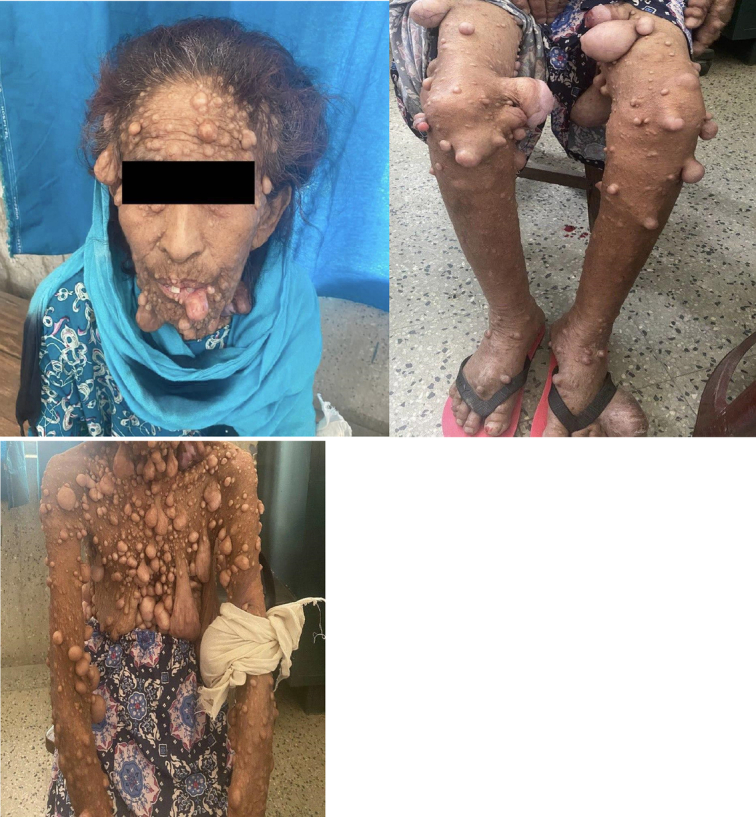
Cutaneous neurofibromas.

**Figure 2:**
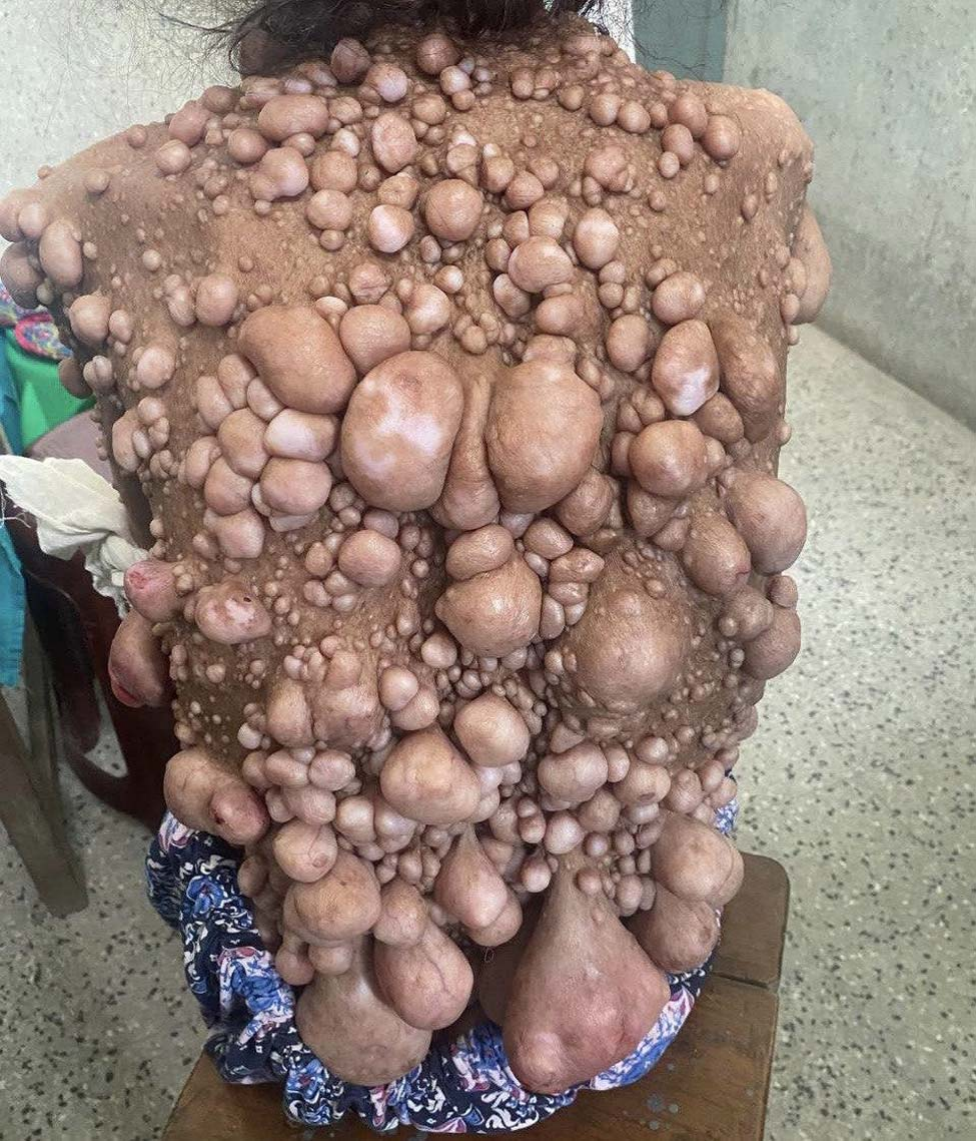
Many soft neurofibromas are present on the trunk, with some having stalks (plexiform neurofibroma).

**Figure 3:**
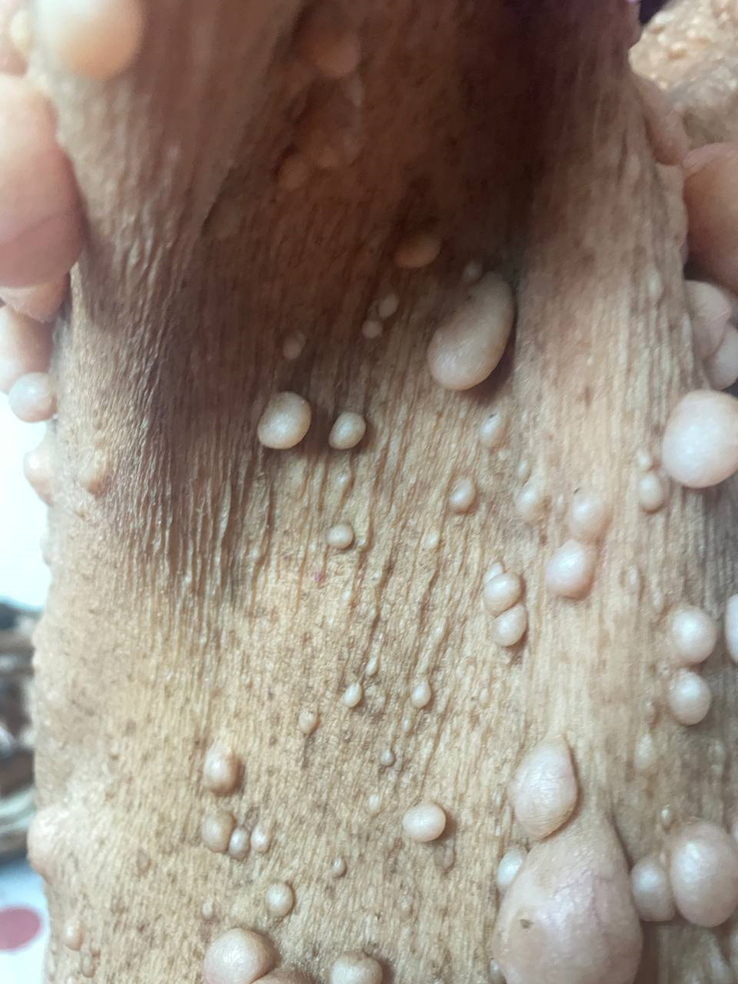
Axillary freckling.

The dermatological examination revealed numerous soft neurofibromas on the patient’s skin, varying in size from small millimeters to larger centimeters, with some having stalks. These lesions were most prominent on the face, trunk, and limbs. In addition, multiple café-au-lait spots larger than 1.5 cm in diameter were observed, along with freckling in the axillary regions. Figures associated with these findings are as follows:(Figs. 1–3).

**Figure 4:**
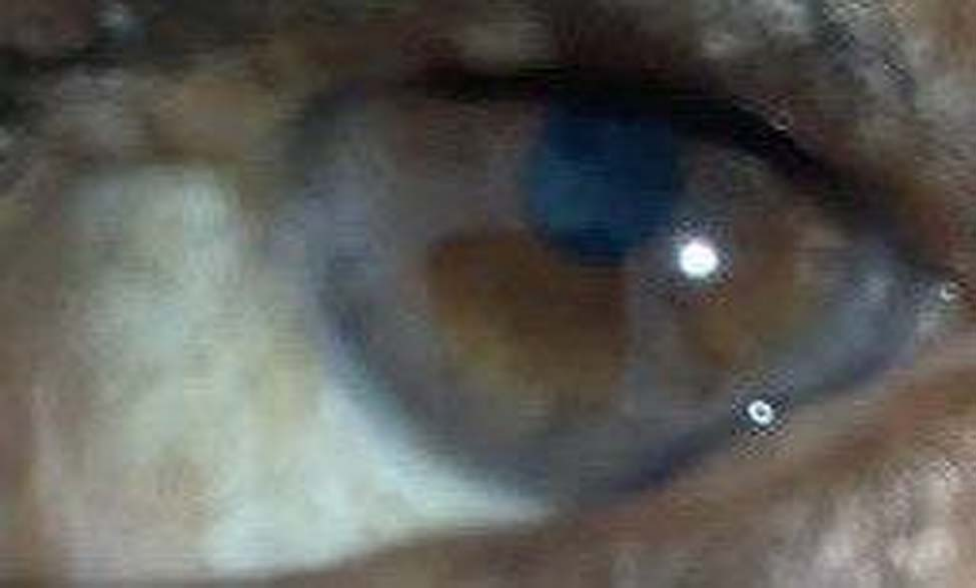
Lisch’s nodules.

## Ophthalmological status

The ophthalmological examination revealed fibromas on both eyelids, measuring about 1.5 cm, causing partial ptosis. Additionally, Lisch nodules were identified on the irises of both eyes, although they did not significantly impact vision Fig. 4.

## Laboratory investigations

Laboratory data revealed normal values in standard tests. The detailed laboratory findings are organized in the tables below: (Table 1).

**Table 1:** General laboratory findings

Test	Result	Normal range
Hemoglobin	13.5 g/dl	12–16 g/dl
White blood cell count	7.5×10^9^/l	4–11×10^9^/l
Platelets	250×10^9^/l	150–450×10^9^/l
Blood glucose	90 mg/dl	70–100 mg/dl
Serum creatinine	0.8 mg/dl	0.6–1.2 mg/dl
ALT	22 U/l	7–56 U/l
AST	20 U/l	10–40 U/l
CRP	<5 mg/l	<10 mg/l

ALT: alanine aminotransferase, AST, aspartate aminotransferase; CRP, c-reactive protein.

## Radiological investigations

Radiological evaluations, including X-rays and CT scans, revealed no abnormalities. Given the lack of findings, the imaging results are organized in the table below: (Table 2).

**Table 2:** Radiological findings

Test	Result
Chest X-ray	Normal
CT scan	Normal
MRI	Normal

CT, computed tomography.

Due to significant visual impairment caused by the tumors on her eyelids, the patient was admitted to the Ophthalmologic clinic in Pleven for surgical excision of the tumors. The procedure aimed to improve her peripheral vision and achieve a favorable cosmetic outcome. Histological analysis of the excised tumors confirmed the diagnosis of a benign tumor of nerve tissue. Postoperatively, the patient experienced improved vision, particularly in her right eye, and the upper eyelid structure was successfully restored.

Beyond the management of visual impairment, the comprehensive approach to managing NF1 in this resource-limited setting included several key components. The patient was scheduled for regular follow-up visits to monitor the progression of neurofibromas and any potential complications. Patient education was a crucial aspect, with both the patient and her family being informed about the nature of NF1, its potential complications, and the importance of consistent monitoring. Symptomatic treatment was provided as needed, including pain management and addressing any secondary infections of the neurofibromas. Multidisciplinary care was also emphasized, involving coordination with dermatologists, neurologists, and genetic counselors to address the various facets of NF1 effectively.

## Limitations

This case highlights the challenges and considerations involved in diagnosing and managing NF1 in resource-limited settings. The diagnosis primarily relied on the characteristic clinical features, and basic investigations like blood tests and chest X-rays provided additional support. Management strategies were adapted to available resources, and patient education with a potential referral for advanced evaluation was crucial.

The uniqueness of this case lies in the significant visual impairment caused by neurofibromas on the eyelids, highlighting the necessity of timely intervention to preserve vision. Additionally, the patient’s family history suggests a hereditary pattern, which could provide insights into the genetic aspects of the disease.

In conclusion, this case illustrates the importance of multidisciplinary care in managing complex presentations of NF1. Early diagnosis and intervention can significantly improve the quality of life for affected individuals, particularly in preventing complications such as visual impairment. Genetic testing could further confirm NF1, although it was not performed in this resource-limited setting.

I am writing in accordance with the SCARE checklist. In accordance with the SCARE 2023 guideline (Sohrabi *et al*.^10^), the methodology for reporting surgical case details was strictly adhered to in this study.

## Discussion

NF1 is a multisystem disorder with a wide range of clinical manifestations that can complicate its management^11^. The hallmark features of NF1 include multiple café-au-lait spots, neurofibromas, axillary or inguinal freckling, Lisch nodules, and skeletal abnormalities^12^. The development of these features can vary widely among individuals, making each case unique and necessitating a personalized approach to management^13^.

In this case, the patient presented with progressive visual impairment due to neurofibromas on her eyelids, a relatively rare but significant complication of NF1. The visual impairment was primarily caused by the mechanical obstruction of the visual field by the neurofibromas, leading to partial ptosis and restricted peripheral vision^14^. This highlights the importance of early intervention to prevent irreversible visual loss, which is a crucial aspect of NF1 management^15^.

The patient’s family history of multiple café-au-lait spots suggests a hereditary pattern of NF1, consistent with its autosomal dominant inheritance^16^. Genetic testing, although not performed in this case due to resource limitations, could have provided definitive confirmation of the NF1 diagnosis and insights into the specific mutation involved^17^. Genetic counseling is essential for patients and their families to understand the inheritance pattern and the risks of transmission to offspring^18^.

The management of NF1 in this case was adapted to the resource-limited setting, emphasizing the importance of regular follow-up visits, patient education, and symptomatic treatment^19^. The multidisciplinary approach involving dermatologists, neurologists, and genetic counselors was crucial in addressing the various aspects of NF1 and providing comprehensive care^20^. This approach aligns with current recommendations for the management of NF1, which advocate for regular monitoring and multidisciplinary care to manage the diverse manifestations of the disease effectively^21^.

The surgical excision of the neurofibromas on the patient’s eyelids was a pivotal intervention that improved her vision and cosmetic appearance^22^. Histological analysis confirmed the benign nature of the tumors, consistent with the typical presentation of neurofibromas in NF1^10^. Postoperative follow-up was essential to monitor for recurrence and manage any potential complications, underscoring the need for ongoing care in NF1 patients ^23^.

This case also underscores the challenges faced in resource-limited settings, where access to advanced diagnostic tools and specialized care may be restricted. The reliance on clinical features and basic investigations for diagnosis highlights the need for practical and adaptable management strategies^24^. Patient education and involvement in care decisions are crucial in such settings to ensure adherence to follow-up and treatment plans.

## Conclusion

This case report highlights the complexities of diagnosing and managing NF1 in a resource-limited setting. The significant visual impairment due to neurofibromas on the eyelids emphasizes the necessity for timely intervention to preserve vision and improve quality of life. Multidisciplinary care and patient education were crucial components of the management strategy. This case underscores the importance of adaptable management approaches and highlights the potential benefits of genetic testing for comprehensive care in NF1 patients.

## Ethical approval

Not applicable.

## Consent

Informed consent was taken from the patient to publish this case report.

## Source of funding

No funding was obtained for this study.

## Author contribution

P.A. and N.B. provided with data and materials from the archive and their notes. P.A. and N.B. wrote the manuscript, collected the images and put them in perspective according to the timeline of the case. P.A. reviewed the manuscript and did final editing.

## Conflicts of interest disclosure

The authors declare no conflict of interest.

## Research registration unique identifying number (UIN)

This is a cross-sectional involving a human subject, so registration of research study was done.

1. Registry used: Researchregistry.com.
2. Unique Identifying number or registration ID: researchregistry10455.
3. https://www.researchregistry.com/browse-the-registry#home/.

## Guarantor

Pratik Adhikari is the guarantor of the study.

## Data availability statement

The datasets supporting the conclusions of this article are included within the article.

## Provenance and peer review

Not commissioned or externally peer-reviewed.
